# Exploring cultural competence barriers in the primary care sexual and reproductive health centres in Catalonia, Spain: perspectives from immigrant women and healthcare providers

**DOI:** 10.1186/s12939-024-02290-5

**Published:** 2024-10-09

**Authors:** Jone G. Lurgain, Hakima Ouaarab-Essadek, Khadija Mellouki, Sumaira Malik-Hameed, Andleed Sarif, Laia Bruni, Valentina Rangel-Sarmiento, Paula Peremiquel-Trillas

**Affiliations:** 1 Department of Public Health, Environments and Society, London School of Hygiene and Tropical Medicine, Keppel Street, London, WC1E 7HT UK; 2 Community & Public Health Team (ESPIC), Centre for International Health and Infectious Diseases, Drassanes-Vall d’Hebron, Carrer de Sant Oleguer, 17, Barcelona, 08001 Spain; 3 Cancer Epidemiology Research Programme, Catalan Institute of Oncology, Av Gran Via 199-203, L’Hospitalet de Llobregat, Barcelona, 08908 Spain; 4 Bellvitge Biomedical Research Institute – IDIBELL, Av Gran Via 199-203, L’Hospitalet de Llobregat, Barcelona, 08908 Spain; 5 Consortium for Biomedical Research in Epidemiology and Public Health, CIBERESP. Carlos III Institute of Health, Av De Monforte de Lemos 5, Madrid, 28029 Spain; 6 Faculty of Nursing and Health Sciences, University of Barcelona, L’Hospitalet de Llobregat, C/Feixa Llarga s/n, Barcelona, 08907 Spain

**Keywords:** Cultural competence, Health inequities, Immigrants, Sexual and reproductive health, Health service research

## Abstract

**Background:**

Immigrant populations, especially women, continue facing challenges in accessing quality healthcare, particularly sexual and reproductive health services (SRH). Poor cultural competent health systems contribute to communication challenges between immigrant women and healthcare providers perpetuating health disparities. This exploratory study describes these communication barriers from the perspective of Moroccan and Pakistani immigrant women and healthcare providers within the Catalan health system and its implications to ensure an equitable provision of SRH services.

**Methods:**

An exploratory-descriptive qualitative study was conducted in various municipalities of Barcelona with high concentration of immigrants. Eight focus groups (*N* = 51) and semi-structured interviews (*N* = 22) with Moroccan and Pakistani immigrant women were combined with key informant interviews (*N* = 13) with healthcare professionals. Thematic analysis and data triangulation were performed primarily using an inductive approach.

**Results:**

Language barriers and cultural differences in health needs, expectations, care-seeking behaviours and understanding of quality healthcare provision hindered the ability of immigrant women and providers to interact effectively. Limited availability of intercultural mediators and inadequate cultural competence training opportunities for health staff were also identified. Findings suggest a lack of minority representation in the Catalan health workforce and leadership roles.

**Conclusion:**

This study reinforces the evidence of persistent inequities in accessing healthcare among immigrant populations by focusing on the cultural competence barriers of the Catalan health system in the provision and access to SRH services. The regularization of adequately trained intercultural mediators, quality training in cultural competence for health staff and a commitment to increase workforce diversity would contribute to improve intercultural communication between immigrant patients and providers. An urgent call to action in this direction is needed to ensure an equitable access to SRH services among immigrant women.

**Supplementary Information:**

The online version contains supplementary material available at 10.1186/s12939-024-02290-5.

## Background

Despite efforts made over the last two decades, evidence of disparities between immigrants and non-immigrants in accessing healthcare services persist across Europe [1–3]. Poor cultural competent health systems contribute to communication challenges between immigrant patients and healthcare professionals limiting an equitable access and use of healthcare services [4, 5]. For instance, poor patient-provider communication can negatively affect the quality of care, satisfaction, treatment compliance and, ultimately, patients’ health outcomes [6, 7]. Communication is especially challenging in the provision of sexual and reproductive health (SRH) services due to the sensitivity of the topic. In this context, communication barriers undermine immigrant women’s right to benefit from SRH care and to make informed decisions concerning their health [5, 8]. Addressing these communication barriers between immigrant patients and healthcare professionals is, therefore, imperative and urgent.

Intercultural communication in healthcare is the interaction between healthcare professionals and patients of different cultural groups (international, interfaith, interethnic, interracial) to reach an understanding, build a shared reality and establish a satisfactory relationship [9, 10]. It requires verbal and non-verbal communication skills, cultural sensitivity, constructive emotional expression, cultural knowledge and adaptability [11]. Competence in intercultural communication is also considered a self-reflective practice that can be inculcated, trained and achieved [12]. Healthcare professionals’ intercultural communication skills help to provide a comfortable environment for discussion and involvement of patients in the decision-making process, as well as for trust [13], resulting ultimately in better health outcomes [14]. Competence in intercultural communication is, therefore, critical to ensuring quality healthcare delivery for immigrant populations and reduce health inequities.

The concept of cultural competence goes beyond healthcare providers’ intercultural communication skills and also involves the ability of organizations to effectively deliver healthcare services that meet the social, cultural and linguistic needs of patients [15], which is particularly crucial when serving immigrant populations [16–19]. The deployment of intercultural mediators -whose role is to reduce linguistic but also cultural barriers between patients and providers [20]- and the provision of training for health staff are common cultural competence interventions adopted by health systems to improve accessibility and effectiveness of health services for immigrant populations [17, 20–23]. Studies have shown that intercultural mediators can increase the uptake of preventive measures [24] and patient and provider satisfaction [25, 26], reduce adverse events [27], as well as rates of unnecessary examinations, hospitalisations and costs [27, 28]. Despite these positive effects, intercultural mediator services are scarce in many European countries [29]. Additionally, cultural competence training is seldom included in the healthcare professionals’ curriculum, and they consequently bear the responsibility of filling the gap and developing their own cross-cultural competences [30–34].

In Spain, significant immigration flows began at the turn of the millennium when the non-native population doubled in just one decade, a growing tendency which has continued until now [35, 36]. Catalonia was one of the regions that witnessed the largest influx of immigrants and, currently, its foreign population reaches 1.2 million (16.3% of the Catalan population) [37]. The largest non-EU majority groups, excluding immigrants from Latin America, come from Morocco (18.5% of the migrant population), China (4.9%) and Pakistan (4.3%) [37], where most citizens’ first language is neither Spanish or Catalan, and cultural differences are notable with respect to the host country [38].

Up to date, a very few studies have addressed cultural competence barriers in the provision of SRH services in the Spanish and Catalan contexts [39, 40]. Therefore, this study aims to fill this gap by describing the communication challenges between immigrant patients and providers in the context of the Catalan health system. Using the case of Moroccan and Pakistani immigrant women, the study explores the potential impact of these barriers to access SRH services and potential strategies to improve the Catalan health system’s cultural competence. The perspectives of immigrant women and healthcare providers are presented together in a constructive way and recommendations are provided for policy makers’ and practitioners’ consideration.

## Methods

### Study design

An exploratory-descriptive qualitative research design [41] was used to explore cultural competence barriers in the provision of care within the primary care SRH centres (referred to as ASSIR) in Barcelona province. We focused on communication barriers between Moroccan and Pakistani immigrant women and healthcare providers in accessing SRH services, and particularly cervical cancer screening since the study was embedded in a larger organised population-based cervical cancer screening implementation program in Catalonia, Spain. This qualitative design enabled us to gain an in-depth understanding of what happens before, during and after the medical encounter and reasons for communication barriers as perceived by Moroccan and Pakistani women and healthcare providers.

### Sampling and recruitment strategies

Immigrant women and healthcare providers were selected using maximum variability sampling: convenience, purposive and snowball sampling techniques were combined based on age, length of stay in Spain, educational background or healthcare role. Inclusion criteria for immigrant women were determined by their country of origin (Morocco and Pakistan), age at migration (16 years or older, thus not exposed to the public Spanish education system) and their eligibility to the cervical cancer screening program in Catalonia (aged 25 or turning 25 in the year of the study, up to 65 and having a cervix). The main inclusion criteria for healthcare professionals were that they needed to be working actively in the primary care SRH centres and interacting directly with these two groups of immigrants. No exclusion criteria were established for healthcare professionals, but we ensured that providers reported at least ten consultations per week with immigrant patients from Morocco and/or Pakistan. Half of healthcare professionals reported that at least 50% of their patients were immigrants. The final sample size was determined when data saturation was achieved, indicating that additional interviews no longer provided new insights into the key dimension of the study.

We recruited Moroccan and Pakistani participants in neighbourhoods of Barcelona city, Sant Adrià del Besòs, Terrassa and L’Hospitalet de Llobregat. These were chosen because they represent different health districts, have a high proportion of Moroccan and Pakistani immigrants and include urban and semi-urban residential areas.

We recruited a wide range of healthcare professionals, such as community health workers, nurses, midwives, gynaecologists, all of whom worked in primary care SRH centres located in the same areas where most women participants were recruited. An intercultural mediator was also included, as these professionals greatly contribute in the provision of care for immigrants.

### Data collection

Study fieldwork was performed between July and December 2022. A total of 8 focus group discussions (FGDs) (4 [all in Darija] with 24 participants from Morocco and 4 [all in Urdu] with 27 participants from Pakistan), 22 semi-structured interviews (SSIs) (12 [7 in Darija and 5 in Spanish] with Moroccan and 10 [5 in Urdu, 1 in Punjabi and 4 in Spanish] with Pakistani women) and 13 key informant interviews (KIIs) [all in Spanish] with healthcare providers were conducted.

Topic guides for FGDs and SSIs with immigrant women were designed based on a literature review on barriers and facilitators to cervical cancer screening among immigrant populations conducted for a broader study mentioned above. Questions covered individual (i.e. lack of language skills), interpersonal (i.e. social influences and cultural norms) and health system (i.e. interpreting and translation services) level factors. FGDs were moderated by a nurse (HOE) and a community health worker (SMH) with the same cultural and linguistic background as participants, and the first author (JGL) and two research assistants (RAs) (KM and AS), second-generation immigrant university students, conducted the SSIs. FGDs were conducted in community and religious centres (e.g. mosques) and the interviews were conducted in places chosen by the participants (e.g. women’s and interviewers’ home, religious centres). Further details of the data collection process have been reported elsewhere [42].

Interviews with healthcare providers explored their views and experiences providing SRH services, and specifically cervical cancer screening, to immigrant women. The overall goal was to understand how these women access healthcare, their preventive health practices, and their engagement with SRH services from the perspective of those who directly work with immigrant populations. These KIIs with healthcare providers also aimed to identify potential cultural and religious barriers and discuss strategies to increase participation in cervical cancer screening programmes, including the potential acceptance of HPV self-sampling. The first author (JGL) carried out all the interviews with healthcare professionals. These took place in the providers’ workplace, except three interviews which were conducted either at the interviewee’s home (*N* = 2) or via phone (*N* = 1).

### Data analysis

The RAs (KM and AS) transcribed women’s audio recordings from Darija and Urdu directly into Spanish and English, respectively, with minor editing to improve readability. The first author (JGL) and a co-author (VRS, physician and public health student) transcribed healthcare providers’ audio recordings into Spanish. We conducted a social constructionist thematic analysis [43, 44] using mainly an inductive (data-driven) approach, although we used some predetermined themes (deductive approach), such as language limitations. We developed preliminary codebooks, using pen-and-paper and Microsoft Office Word and Excel, in which themes and sub-themes were defined [45]. JGL, VRS and PPT independently coded two transcripts from each type of dataset (FGDs and SSIs with immigrant women and KIIs with healthcare professionals) and after discussion, agreement on a codebook (one for immigrant women and one for healthcare providers) was reached. Transcripts were uploaded into the qualitative data management software ATLAS.ti 23 [46] and the first author (JGL) developed the final codebooks incorporating new emerging codes or removing those which were not relevant. For validation of themes and sub-themes, the above researchers coded additional transcripts using the final codebooks. FGD and SSI data obtained from Moroccan and Pakistani women and data from the KII with the healthcare providers were then analysed separately with the assistance of ATLAS.ti 23. See final codebooks with themes and sub-themes and examples of supporting quotes in Supplementary materials 1 and 2.

After coding and analysing the two datasets from immigrant women and healthcare providers separately, a data triangulation process was performed following a ‘complementary’ approach: searching to describe a problem (e.g. cultural competence barriers to access SRH services) from different perspectives [47]. Specifically, the triangulation process was guided by the cultural competence framework proposed by Betancourt et al. (2003) [15], which allowed us for an overall interpretation of the findings. This framework identifies three major levels of healthcare at which cultural competence barriers occur that contribute to health disparities: (1) clinical barriers, which occur when sociocultural differences in the interaction between patients and providers are not fully accepted, appreciated, explored or understood; (2) structural barriers, which refer to the availability of trained intercultural mediator services or culturally/linguistically appropriate health education materials, and (3) organizational barriers, which pertain to the representation of racial/ethnic minorities in the healthcare workforce and leadership roles. A summary of the main themes and sub-themes is provided in Table 1 and the triangulation tool is provided in Supplementary material 3 with examples of supporting quotes.

**Table 1:** Summary of cultural competence barriers in the provision of SRH services in the Catalan health system using Betancourt et al.’ (2003) framework

Themes	Sub-themes
Theme 1:	
Clinical barriers	• Lack of language skills • Perceived discriminatory attitudes • Time pressure in the provision of care • Cultural taboos (e.g. offering certain SRH services) • Limited patient informed consent and confidentiality
Theme 2	
Structural barriers	• Limited availability of intercultural mediators • Limited and underused translation services and materials • Lack of adequate cultural competence training for health staff
Theme 3	
Organisational barriers	• Low minority representation in the healthcare workforce (e.g. nurses) • Low minority representation in leadership roles
*Note that some of the clinical and structural barriers could also be organisational.	

### Ethical considerations

The study was approved by the Research Ethics Committees of the London School of Hygiene & Tropical Medicine (26186), Bellvitge University Hospital (PR 140/22) and Vall d’Hebron University Hospital (PR(AG)317/2022). Each participant was provided with comprehensive information about the study and gave written informed consent prior to data collection. Immigrant women were compensated with a public transportation 10-trip pass to ensure that participation did not impose a financial burden on them (transport to FGDs’ and SSIs’ locations) and to acknowledge their contribution to the research. Healthcare professionals did not receive any compensation.

## Results

### Participants’ socio-demographic characteristics

The Moroccan and Pakistani participants’ ages ranged from 24 to 65 years. The majority (82.2%) were married and nearly all Pakistani women (97.3%) had children, compared with 77.8% of Moroccan women. The primary reason for migration was family reunification. Despite most women (80.0%) of both groups having lived in Catalonia for over 5 years, up to 63% of them (52.8% Moroccan and 73% Pakistani) reported needing a translator during medical visits. A similar percentage was found among women who had arrived in the country (less than 5 years ago). Moroccan women were more likely to speak Spanish or Catalan at home than Pakistani women (33% vs. 5%). A total of 72.6% of participants reported having been screened for cervical cancer at least once in their lifetime. Women’s detailed socio-demographic information can be found in Table 2.

Healthcare providers’ ages ranged from 29 to 59 years. All of them were female, except for a gynaecologist and a community health worker. Furthermore, eight healthcare professionals were Spanish (mainly from Catalonia) and bilingual (Spanish/Catalan) and, five were originally from either Morocco or Pakistan and one of them also bilingual (Spanish/Catalan) (Table 3).

**Table 2:** Women’s socio-demographic characteristics by country of origin (*N* = 73)

	Total participants		Morocco		Pakistan	
	*N*	(%)*	*N*	(%)*	*N*	(%)*
**Participants** ^**1**^	73	*(100%)*	36	*(49.3%)*	37	*(50.7%)*
**Age median (IQR)** ^**2**^	42	*(35–48)*	39	*(33–46)*	40	*(34–47)*
**Age groups**						
24–34 years	18	*(24.7%)*	7	*(19.4%)*	11	*(29.7%)*
35–44 years	28	*(38.4%)*	13	*(36.1%)*	15	*(40.5%)*
45–54 years	19	*(26.0%)*	10	*(27.8%)*	9	*(24.3%)*
55–65 years	8	*(11.0%)*	6	*(16.7%)*	2	*(5.4%)*
**Level of studies**						
No studies	9	*(12.3%)*	7	*(19.4%)*	2	*(5.4%)*
Primary school	15	*(20.5%)*	9	*(25.0%)*	6	*(16.2%)*
Secondary school	25	*(34.2%)*	14	*(38.9%)*	11	*(29.7%)*
Vocational training	3	*(4.1%)*	2	*(5.6%)*	1	*(2.7%)*
University	21	*(28.8%)*	4	*(11.1%)*	17	*(45.9%)*
**Marital status**						
Single	4	*(5.5%)*	4	*(11.1%)*	0	*(0.0%)*
Married	60	*(82.2%)*	26	*(72.2%)*	34	*(91.9%)*
Separated or divorced	7	*(9.6%)*	5	*(13.9%)*	2	*(5.4%)*
Widowed	2	*(2.7%)*	1	*(2.8%)*	1	*(2.7%)*
**Children**						
Yes	64	*(87.7%)*	28	*(77.8%)*	36	*(97.3%)*
**Time since migration to Spain**						
< 2 years	7	*(9.6%)*	2	*(5.6%)*	5	*(13.5%)*
2–5 years	11	*(15.1%)*	8	*(22.2%)*	3	*(8.1%)*
6–10 years	19	*(26.0%)*	4	*(11.1%)*	15	*(40.5%)*
> 10 years	36	*(49.3%)*	22	*(61.1%)*	14	*(37.8%)*
**Reason of migration**						
Economic	1	*(1.4%)*	1	*(2.8%)*	0	*(0.0%)*
Family reunification	62	*(84.9%)*	27	*(75.0%)*	35	*(94.6%)*
Tourist/student visa	7	*(8.2%)*	6	*(16.7%)*	1	*(2.7%)*
Not reported	3	*(2.7%)*	2	*(5.6%)*	1	*(2.7%)*
**Languages most spoken at home** ^**3**^						
Spanish or Catalan^4^	14	*(19.2%)*	12	*(33.3%)*	2	*(5.4%)*
Arabic (Darija)	31	*(42.5%)*	31	*(86.1%)*	0	*(0.0%)*
Urdu	33	*(45.2%)*	0	*(0.0%)*	33	*(89.2%)*
English	5	*(6.8%)*	1	*(2.8%)*	4	*(10.8%)*
French	3	*(4.1%)*	3	*(8.3%)*	0	*(0.0%)*
Other^5^	8	*(11.0%)*	3	*(8.3%)*	5	*(13.5%)*
**Spanish language skills**						
I always need a translator	20	*(27.4%)*	8	*(22.2%)*	12	*(32.4%)*
Most of the times I need a translator	8	*(11.0%)*	3	*(8.3%)*	5	*(13.5%)*
Sometimes I need a translator	11	*(15.1%)*	6	*(16.7%)*	5	*(13.5%)*
I do not need translator at all	27	*(37.0%)*	17	*(47.2%)*	10	*(27.0%)*
**Public health insurance**						
Yes	68	*(93.2%)*	34	*(94.4%)*	34	*(91.9%)*
No	2	*(2.7%)*	0	*(0.0%)*	2	*(5.4%)*
**Cervical cancer screening status**						
Ever screened	53	*(72.6%)*	27	*(75.0%)*	26	*(70.3%)*
Never screened	14	*(19.2%)*	7	*(19.4%)*	7	*(18.9%)*
I don’t know what screening is	4	*(19.2%)*	0	*(0.0%)*	4	*(10.8%)*

CC: cervical cancer; IQR: interquartile range

^*^ Column percentages; percentages may not add due to missing values

^1^Percentages correspond to row percentages

^2^Median and IQR were used as variable age was not normally distributed

^3^Percentages were calculated among the total participants for each language, as multiple options could be selected in the socio-demographic questionnaire

^4^Twelve women spoke Spanish at home. One woman reported only Catalan and another reported Catalan and Spanish as languages most spoken at home

^5^Includes Riffian (N = 3, Moroccan women) and Kashmiri and Punjabi (N = 1 and N = 4, respectively, among Pakistani women)

**Table 3:** Characteristics of healthcare providers interviewed

Key informant interviews (*N* = 13)	Sex	Age	Country of origin	Occupation	Healthcare experience (years)
KI01	F	46	Morocco	Coordinator nurse	18
KI02	F	55	Spain	Midwife / Management role	30
KI03	F	33	Spain	Gynaecologist	7
KI04	F	45	Pakistan	Community Health Agent	8
KI05	F	59	Spain	Midwife	35
KI06	F	31	Spain	General Practitioner	5
KI07	M	49	Morocco	Community Health worker	10
KI08	F	32	Morocco	Intercultural mediator	9
KI09	M	36	Spain	Gynaecologist	10
KI10	F	30	Spain	Gynaecologist	4.5
KI11	F	29	Spain	Midwife	6
KI12	F	50	Spain	Midwife	26
KI13	F	29	Spain	Midwife	5

### Cultural competence barriers

Qualitative findings from the FGDs and SSIs with immigrant women, as well as from the interviews with healthcare professionals are presented below and organised by each of the cultural competence barriers identified in the framework proposed by Betancour et al. (2003) [15].

### Clinical barriers

#### Lack of language skills

We identified the inability to understand and speak either of the two official languages in Catalonia (Spanish/Catalan) as a barrier among Moroccan and Pakistani women regardless of their length of stay in the country. According to healthcare providers’ perceptions, language barriers appeared more prevalent among Pakistani than Moroccan women.

Women from both countries often depended on the availability of their husbands, family members and friends to arrange appointments or to receive information. For instance, when their male partners could not accompany them, some women preferred to skip or postpone medical appointments until someone else was available. Whereas Pakistani women tended to visit the doctor with their husbands, Moroccan participants counted also on friends and even on other compatriots they met while waiting in the primary care centre for appointments.

*“Many (Pakistani) women don’t speak Spanish properly and they depend on their husbands’ schedules to go to the doctor*,* and husbands work the whole day from Monday to Friday*,* so that’s why women don’t want to come” PC01 (Pakistani woman)*.

*“I prefer to go to the doctor with someone who I know to help me with translation. I think I’m more comfortable. In the past my neighbour used to come with me” MC06 (Moroccan woman)*.

Whilst the support of the husband and other family members and friends seemed to attenuate the language barriers of women, this did not guarantee that they received accurate information about SRH services. Healthcare providers reported inadequate and incomplete translations by male partners and family members:

*“Sometimes I see the husband explaining her wife what the doctor is saying*,* but not all*,* some just say a few things because they think the wife doesn’t need to know everything” KI04 (Pakistani community health worker)*.

*“The first and most important thing is to make sure that women understand the information we provide*,* because we often give them the information through a third party [referring to the husband] who is not transmitting the information properly. It’s crucial to have a mediator or a person committed who can explain all of this*,* otherwise women won’t come to get screened” KI05 (female midwife)*.

Immigrant women and healthcare providers mentioned having personal strategies to overcome language barriers. Some women managed to communicate with providers using body language, through mobile phone conversations with family members or through paper notes, as this provider explained:

*“They usually come with their own resources*,* like a mobile phone and someone on the other side of the phone line who does the translation*,* or a friend*,* or a neighbour… and sometimes they bring me a paper with the health issues or questions written by their partner (in Spanish) and I respond to them by hand on the same paper. I also explain to them that they need to come with a person who can translate*,* otherwise it’s very complicated for her to understand the treatment” KI12 (female midwife)*.

Body language, visual materials (e.g. pictograms), physical objects (e.g. plastic uterus), translated forms and Google Translator were resources used by midwives and gynaecologists to overcome language barriers:

*“We manage with our body language; it’s true that using gestures*,* we and Moroccan women understand each other quite well*,* but non-verbal communication with Pakistani women is more difficult*,* because their body language*,* gestures are different to ours. I’m always afraid of doing a gesture that can appear offensive” KI02 (female midwife)*.

*“We used to have some pictograms in different languages*,* but what I usually do is to give the information with drawings and sometimes with images as they help them to visualize a little bit what and where is the health issue” KI03 (female gynaecologist)*.

They also emphasized the importance of expressing empathy and building rapport with immigrant patients through eye contact and using some words in their local languages to improve communication, as this female midwife explained: *“I have learned a couple of words in Arabic and I usually use them with Maghrebi women. They can see that I make efforts to facilitate communication and I create some empathy” KI05 (female midwife).* The use of ‘third’ languages, such as English and French, was also mentioned by women and healthcare providers: *“I speak a little French*,* sometimes I ask Moroccan patients if they speak French*,* if they say ‘yes’*,* then we manage in French” KI09 (male gynaecologist).*

#### Perceived discriminatory attitudes

While immigrant women considered the Catalan health service to be good overall, even better than in their own countries, and healthcare providers to be “caring”, some women felt they did not receive the care they expected. Several participants reported a lack of empathy and unhelpful attitudes from the health staff, especially clinic receptionists, but also some healthcare providers. Women from both Moroccan and Pakistani origin reported feelings that health staff were racist and held discriminatory attitudes against them when they could not communicate properly in Catalan or Spanish, for instance, to book a medical appointment. This deterred some women from seeking healthcare services in Catalonia and instead, seeking care in their own countries:

*“A few years ago*,* I couldn’t speak Spanish and I couldn’t find anyone who could accompany me to the midwife consultation. My husband worked in Portugal. Perhaps it was my responsibility to bring someone and be able to answer her questions… She spoke to me very aggressively. I can’t forget it” MG202 (Moroccan woman)*.

*“Generally*,* they [healthcare providers] are kind*,* but there are receptionists in the health centres who don’t have any sensitivity when they attend a person who can’t speak properly in Spanish or Catalan. Instead of being empathetic and try to communicate or ask for help*,* they just ask ‘what are you saying?’*,* then*,* this person won’t come back. This is what happens with Pakistani women*,* they prefer to stay at home or wait to go to the gynaecologist in their country and pay*,* rather than face these unpleasant situations. Honestly*,* we prefer to pay [back home] and avoid taking an appointment here” PC05 (Pakistani woman).*

Immigrant women also reported negative and discriminatory attitudes from healthcare providers, especially clinic receptionists, when they requested or expressed their preference for a female professional to undertake gynaecology examinations, deterring them from requesting a same sex provider in the Catalan health system:

*“There is a Moroccan woman in my neighbourhood who requested a female doctor in her surgery and up to now she has been having troubles just because she wanted a female doctor (…) Sometimes the receptionists in the health centre don’t like that an immigrant woman requests a female doctor…they think she needs to accept what is available*,* that’s it*,* they think that immigrants do not have rights and can’t demand anything” MC01 (Moroccan woman)*.

*“I had an experience here*,* a woman who recently came from Pakistan*,* she requested a female doctor*,* and the receptionist started to argue and say: ‘that’s the problem with you Pakistanis’ and then the woman didn’t get an appointment until three months later and the scan was in another hospital far from there and the husband asked to get the appointment in their health centre but the receptionist ignored it” PG203 (Pakistani woman)*.

We observed that lack of culturally sensitive policies or clear guidelines to address cultural conflicts led to some confusion concerning the right of immigrant women to request a healthcare professional of the same sex. Most providers interviewed understood Moroccan and Pakistani women´s preferences for female doctors and stated that they should have the right to request it. However, two gynaecologists raised concerns that choosing the sex of the health professional would contradict the gender equity and non-discrimination policies of the Catalan health system:

*“You have the right to have a healthcare provider*,* to be attended*,* but you can’t choose whether the doctor is white or black*,* or whether the doctor is female or male*,* because it’s considered discriminatory. In the private health system*,* yes*,* you can choose whatever you want*,* but in the public health system*,* not” KI09 (male gynaecologist)*.

*“They can’t officially request a female doctor just like you can’t request a white gynae or a heterosexual gynae … It’d bother me if someone doesn’t want me to visit her just because I’m a woman. I know that there is a cultural barrier*,* but it annoys me that a professional can be judged because of his/her gender (…) Obviously*,* if the woman has suffered a sexual aggression*,* all of us would be empathetic [to her preference for a female provider]” KI10 (female gynaecologist)*.

#### Time pressure

Even when immigrant women possessed basic or everyday language capacity, some felt they were not being listened to properly as health providers did not give them sufficient time to explain themselves:

*“Despite my broken Spanish*,* I try to explain the doctor my problems in detail*,* but she always cuts me off and doesn’t give me enough time to express myself. It’s like they work under lot of pressure*,* and since they have lots of patients*,* they attend you quickly” MC11 (Moroccan woman)*.

Healthcare providers confirmed this impression, explaining they have limited time allocated for each patient. Time pressure was even stronger when seeing immigrant patients with language difficulties, low literacy and lack of knowledge of the Catalan health system. This led providers to feel more overloaded and to express feelings of frustration and exhaustion:

*“These visits are difficult and require more time… The time factor is like a sword stuck in your back. Sometimes you need to spend more time with these women to make sure they understand why we are doing these tests [cervical cancer screening tests]. (…) After so many years*,* I’m at a point where I feel exhausted (.) I’m constantly gesticulating to try to reach and communicate with these women; it’s exhausting” KI05 (female midwife)*

*“Sometimes they don’t understand the treatment*,* some of them don’t know how to navigate the health system or how to get the public health insurance (…) Some have low education and many times they come with medical consultations*,* basic issues*,* such as a headache*,* that other people wouldn’t be worried about [meaning additional burden]” KI11 (female midwife)*.

#### Cultural taboos

We identified cultural differences between women and healthcare providers’ beliefs and perceptions regarding the use of certain SRH services and the context in which they would discuss them. Addressing women’s health issues, such as menstruation, in front of male relatives (e.g. husbands, sons) and offering specific SRH services, such as contraception and pregnancy termination services, could be considered offensive or against women’s cultural and religious practices, while for healthcare providers these were common issues addressed with their patients. Cultural differences were also identified regarding certain health examinations, such as cervical cancer screening, at specific times, for example, during Ramadan. A midwife also referred to the difficulties that husbands sometimes found when explaining certain women’s issues:

*“If you are pregnant*,* our religion doesn’t allow us to get abortion*,* you cannot ask the doctor for abortion services… you are pregnant and you need to accept it” MC05* (Moroccan woman).

*“A woman came with her 9 years old son to help her to translate. I asked the child to ask his mum the date of her last menstruation and the child told me that he couldn’t ask this kind of question to his mum… They are difficult situations because culturally, women don’t talk about these topics in front of specific family members [referring to males and children]. For me*,* it was an important barrier because I need this information” KI02 (female midwife)*.

*“For example*,* I had a patient who rejected to be examined because it was Ramadan and she told me it was not allowed during Ramadan. She came three weeks later for the examination” KI03 (female gynaecologist)*.

*“The other day a man came to my consult with his wife who had a vaginal problem and he didn’t know how to express the problem. He told me that she had pain “in the secret”*,* and I couldn’t understand what ‘the secret’ was. He couldn’t explain the problem in a different way. In some cultures*,* men find it difficult to speak with a woman about these issues” KI11 (female midwife)*.

Healthcare professionals also mentioned difficulties in addressing topics related to sexual health and genitalia. In this, they distinguished between first-generation and second-generation immigrants, perceiving the latter to be more aware of their bodies and willing to ask questions about sexual health, whereas the former were more ‘detached’ from their own bodies and had lower literacy about their own anatomy. This led some healthcare providers to provide basic information about certain tests:

*“Many times we cannot explain much more because otherwise we had to start giving them a class of anatomy in fifteen minutes (…) But it’s true that sometimes I simplify a little bit when it comes to cervical cancer prevention and we just say that we’ll check that ‘everything is ok’” KI11 (female midwife)*.

#### Informed consent and confidentiality

While immigrant women considered their husbands and other relatives as the most important social support in the host country, the health professionals sometimes found them more of an obstacle during health consultation. For instance, some professionals expressed concern about whether immigrant women made informed decisions about their SRH: *“Many times I have the impression that it’s the husband who makes the decisions for them. For example*,* I ask him to translate and he just makes a very short translation and then he basically orders her to take off her underwear for the vaginal examination. But I need her to understand me*,* I need him to translate well and her to make the decision” KI02 (female midwife).*

Despite this informed consent issue, a midwife emphasized the importance of establishing a good relationship with husbands due to their role as decision-makers. However, she pointed that culturally this is a challenging goal:

*“…It’s really hard*,* but it’s like this. If you have some empathy with the husband*,* this woman will come back*,* because we can’t build rapport with some women*,* they don’t speak any word in Spanish” KI05 (female midwife)*.

Similarly, healthcare professionals felt that the presence of a family member reduces confidentiality and provider-patient privacy, something that is a foundation of how healthcare professionals understand quality care. For instance, the presence of relatives prevented providers from screening Moroccan and Pakistani women for highly sensitive issues, such as domestic violence or sexually transmitted infections (STIs): *“Of course*,* there are some specific issues we can’t address. For example*,* we have a questionnaire to screen all pregnant women for domestic violence. If her partner comes always to the visit with her*,* we can’t address this. It’s true that you can observe the relationship between them*,* behaviours during the visit*, etc.,* but it’s difficult” KI11 (female midwife)*.

Healthcare providers also reported challenges in ensuring confidentiality when they need to communicate a test result or give an appointment by phone to a woman with language difficulties, and a midwife expressed other ethical challenges they face when immigrant women go to the health centre accompanied by their underaged children to help them with translation. In these circumstances, she explained that professionals skip some explanations regarding the genitals and vaginal examination and give the patient only basic information. Gynaecologists and midwives narrated their strategies to ensure users’ privacy and confidentiality, but all of them agreed that the availability of intercultural mediators would be the most effective support to ensure confidentiality and health informed decisions:

*“When a mediator is available we can ask the partner to get out or the woman can come alone another day and then it’s easier to address these issues [e.g. STIs*,* vaginal problems] with the presence of a mediator” KI11 (female midwife)*.

### Structural barriers

#### Limited availability of trained intercultural mediators

We found disparities in the provision of intercultural mediators across the Catalan health system. Whilst the primary care centres in those neighbourhoods of Barcelona city with the highest concentration of immigrants, such as Raval and Besòs, offered intercultural mediator services in various languages (e.g. Arabic, Urdu, Chinese), in other municipalities with similar volume of immigrants, such as Terrassa and L’Hospitalet de Llobregat, these services were very limited or even non-existent:

*“The availability of intercultural mediators is very important for us. Ten years ago we had the possibility of these services*,* it was a project funded by the Caixa Foundation*,* they trained mediators from different cultures*,* and then unfortunately this service disappeared in almost all primary healthcare centres” KI02 (female midwife)*.

Since mediator services were scarce, informal interpreter services were voluntarily provided by health staff of Moroccan and Pakistani origin, as this gynaecologist explained: *“In the ASSIR unit [primary care SRH centre] we have two auxiliary nurses*,* one from Pakistan and one from Morocco. They are usually working in the maternity ward*,* but sometimes they go to the primary care units to help with translations*,* but it’s not their role*,* they are auxiliary nurses…” KI09 (male gynaecologist).*

We also found that the intercultural mediators working currently in Barcelona are not integrated into the health system. Most of these professionals received training in intercultural mediation in the past and currently they are hired by outsourced companies and work in challenging conditions, as some providers confirmed and a mediator explained: *“The company offers cleaning and security services*,* and now intercultural mediation services. We are hired by this company*,* if we would be directly hired by CatSalud [the Catalan Service of Health*,* the public institution leading health planning and management in Catalonia] our work conditions and salaries would be better“ KI08 (female intercultural mediator).*

Despite the scarcity of intercultural mediators and lack of integration of these professionals within the health system, interviewed healthcare providers agreed that intercultural mediators play an important role in ensuring effective communication and provision of quality care to immigrant patients who lack language skills, especially in the paediatric and primary care SRH centres. They advocated for the regulation of the cost of these services in the Catalan health system and emphasized the importance of equipping these professionals with adequate intercultural mediation training to minimise potential conflicts, as healthcare providers pointed out that these professionals are not mere interpreters, but health mediators with specific training in cultural skills:

*“The difficulty I found in the beginning is that the Moroccan interpreter translated according to her beliefs*,* which was a handicap. For example*,* a young girl with an unwanted pregnancy came and I asked whether she wanted to continue with the pregnancy or interrupt it*,* and the Moroccan interpreter ‘jumped’ into the conversation with a smile telling me ‘we [Muslims] don’t interrupt pregnancies’. I needed to tell her that she only had to translate without judgements or giving opinions. After some time and with more experience*,* we solved this situation. It’s important to train the interpreters [referring to intercultural mediators] and make sure they understand their role” KI12 (female midwife)*.

One healthcare professional added that even when the mediation services are needed and available, these must be offered as an option, referring to the reluctance expressed by some immigrant women to use these services due to a lack of trust. In this sense, a midwife concluded that greater efforts must be made to explain the role of the intercultural mediators and facilitate trustee relationships between these and immigrant patients.

Finally, a midwife highlighted the importance of taking into consideration the gender of the mediators, at least in the primary care SRH centres: *“The women didn’t want him [referring to a male intercultural mediator] to be in the medical consultation*,* because he was a man. They felt uncomfortable with a male interpreter and they were worried that he could know someone in their community” KI02 (female midwife).*

#### Limited and underused translation services

In addition to this insufficient number of intercultural mediators, we found an underutilisation of the available telephone translation service (061 Salut Respon) by providers. They expressed mixed opinions about this service’s effectiveness. Some considered it a good alternative if mediators were not available, but most admitted to not using this service because it was too slow for the limited time assigned per patient and suboptimal to address language and cultural problems. A few healthcare professionals were even unaware of its existence.

*“They put you in contact with a health translator and then you have the conversation with the woman and the interpreter gives you simultaneous translation via phone. I think it’s helpful and ensures anonymity” KI02 (female midwife)*.

*“It’s the slowest thing in the world! I have 15 minutes per visit! It takes ages until they find an available translator*,* you can’t wait for this. We need more resources*,* like mediators. I manage with materials and forms translated into various languages” KI09 (male gynaecologist)*.

The availability of culturally and linguistically appropriate information and education materials seemed also scarce. Healthcare professionals noted that the webpage from the Department of Health (Canal Salut), is translated into English and French in addition to the official languages in Catalonia. They explained that they usually used information and education materials (e.g. questionnaires, forms, health prevention flyers) translated into different languages, although some providers mentioned a lack of translations in Urdu, for instance.

#### Lack of adequate cultural competence training for health staff

The majority of the healthcare professionals interviewed had attended cultural competence training courses at some point in their careers. They found the courses helpful but inadequate, as they were often short, general and lacked interactive activities. Some providers strongly believed that formal and compulsory training on cultural competence should be integrated into the healthcare professionals’ curriculum. Currently, cultural competence training sessions are usually provided by community-based and non-governmental organisations, and lack institutionally funding, relying on the availability of private funds.

*“I miss in our university medical studies this cross-cultural perspective*,* because the view is very ‘Western’ and we need to keep in mind that in other cultures people understand health and wellbeing in different ways. I think the experience working with these populations gives you the knowledge*,* you learn from immigrant patients about their habits*,* cultural beliefs*,* etc. Empathy and cultural sensitivity are the basics” KI06 (female general practitioner)*.

Some healthcare professionals also proposed training administrative staff, particularly receptionists, on migrants’ rights because *“not all health staff know the law and the bureaucratic circuits of the health system. There is confusion among the administrative staff and there are situations in which they may deny health services to immigrants because of lack of knowledge” KI06 (female general practitioner).*

### Organizational barriers

Healthcare providers mentioned that an ideal solution to overcome cultural competence issues would be to have a more diverse health workforce, in this case, more Moroccan and Pakistani nurses, midwives and gynaecologists: *“We had a Chinese gynaecologist and at that time Chinese patients started to come to the clinics like never before” KI02 (female midwife).*

Immigrant women also welcomed the availability of more healthcare staff from their own countries and they thought this would improve communication between immigrant patients and providers. A few Moroccan participants observed an increasing number of pharmacists, nurses, auxiliary nurses and doctors with Arabic language skills: *“He was a Syrian doctor and I liked it*,* he explained me in Arabic and even though my local language is Darija*,* I understood him” MG205 (Moroccan woman).* However, in the leadership roles the minority representation seemed low.

## Discussion

To the best of our knowledge, this is the first study describing cultural competence barriers in the context of the primary care SRH centres in Catalonia, Spain, from the perspective of Moroccan and Pakistani immigrant women and healthcare providers. First, at clinical level, the results indicate that lack of command of the local languages (Spanish and Catalan) and cultural differences in health needs, expectations, care-seeking behaviours and understanding of the provision of quality care hindered the ability of immigrant women and care providers to interact effectively. Second, at structural level, we identified limited availability of intercultural mediator services and translation materials, and inadequate cultural competence trainings available for healthcare staff. Finally, at organisational level, results suggest low minority representation in the health workforce and leadership roles of the Catalan health system.

Lack of knowledge of the host country’s official languages is one of the dominant barriers to obtain accurate health information, and access and use of quality healthcare services [5, 21, 48]. Although language barriers commonly affect first-generation immigrants during the resettlement period [49], our research found that regardless the length of stay in the host country many Moroccan and Pakistani women struggled to communicate with healthcare staff, for instance, to book gynaecological appointments -women often depended on the availability of informal translation services from family or friends-, as well as to convey their health issues and understand healthcare providers’ explanations. These language limitations may have important implications, such as delayed access to timely healthcare, suboptimal care, increased risk of adverse events and dissatisfaction with care received and poor health outcomes, as reported in previous studies with immigrant patients in Canada [49, 50].

The contested issue about the sex of the healthcare provider, especially providing SRH services, has been extensively addressed in the literature [51]. In our study, both immigrant women and healthcare providers reported feelings of perceived discrimination when requesting a female healthcare professional or for being rejected on the basis of gender, respectively, which suggest a lack of culturally sensitive policies to address this type of cultural ‘conflicts’. Guidelines are thus urgently needed to help healthcare providers manage cultural challenges, as other neighbouring countries with longer history of migration (e.g. United Kingdom) already have in place [52]. Feelings of perceived discrimination have been shown in previous research with immigrant women in Finland and Spain, where participants reported “unfriendly” attitudes and poor communication with healthcare staff [53, 54]. In line with our study, in another research conducted in Switzerland, real or perceived discrimination was rarely mentioned by immigrant patients except in relation to experiences at the registration desk prior to clinical appointments [55].

Time pressure was also a relevant factor hindering communication between immigrant patients and healthcare providers. On the one hand, immigrant women reported not being given sufficient time to explain their health problems and, on the other hand, healthcare providers reported that low language skills often meant immigrant patients required longer consultation times and greater efforts to communicate with them. This, along with time constraints due to heavy patient loads, led providers to feeling frustrated and overworked, which was attributed by one gynaecologist to immigrants’ inappropriate use of healthcare services. This perception concerning immigrants’ use of the public healthcare services was also found in previous studies conducted in Catalonia with healthcare providers [39, 56]. However, migrant health literature has demonstrated that immigrant populations’ medical service usage is not as frequent as perceived. For instance, a recent study found that immigrants tend to underuse maternal health services and have an increased risk of poor SRH outcomes [57].

The above findings demonstrate how the intersection of language barriers and lack of sufficient resources (e.g. personnel) hinder the communication between immigrant patients and healthcare professionals. Despite these challenges, we found that both immigrant women and healthcare professionals made efforts to understand each other. Non-verbal communication, such as body language and pictograms, and the use of Google Translator were some personal strategies reported in this and previous studies conducted with migrants in Europe [29] and Southeast Asia [17]. However, while these efforts permitted basic understanding, they did not create the best conditions for trust and facilitation of good patient-provider relationships, as seen in a previous systematic review [58].

Consistent with previous research conducted with immigrant populations in Australia and the US [59, 60], this study confirmed that even when immigrant women started to master Spanish or Catalan languages, communication barriers persisted due to cultural differences. For instance, providers encountered challenges in addressing certain SRH issues (e.g. STI) and offering certain SRH services (e.g. pregnancy termination services), which immigrant patients might find offensive or conflicting with their religious beliefs.

Limitations to ensure confidentiality and obtained informed consent from immigrant women were other concerns raised by healthcare providers, who agreed that a fair deployment of intercultural mediators would help to address the above linguistic and cultural differences. However, we identified significant disparities in the deployment of intercultural mediators across the Barcelona province, with services predominantly available in primary care centres located in specific neighbourhoods within Barcelona city. This limited availability of mediator services aligns with findings from a recent systematic review assessing barriers and facilitators to access interpreter services in European health systems [4]. Where free-of-charge intercultural mediation services exists (e.g. Germany, Croatia, Spain), the cost of these services, however, is not regulated either at national or European levels, leading to inconsistent provision of mediators and perpetuating patient-provider communication challenges, which in turn contributes to health disparities [4]. Therefore, there is also an urgent need to expand and standardize the availability of trained intercultural mediator services within the Catalan health system and across other European regions, particularly in the primary care SRH centres.

As in other studies performed in Pakistan and Finland [61, 62], in our research, healthcare providers expressed a certain amount of anxiety about their lack of cultural competence. In this sense, providers reported insufficient and inadequate cultural competence training opportunities in the Catalan context. Efforts should therefore be made to improve quality and access to this type of training not only for doctors, but also for nurses and other professional health staff, as suggested in another study conducted in Southern Spain with nurses and Moroccan patients [40]. The inclusion of cultural competence in the healthcare professionals’ curriculum needs also to be considered, as previous research advocate [12].

Finally, field observation and key informant consultations suggested that the representation of minorities in the Catalan healthcare workforce is still low. Studies in the US have shown that diversity and inclusion can help organisations improve both patient care quality and financial results [63, 64]. Thus, this is another strategy to consider improving access and provision of quality SRH care to immigrant populations in Catalonia, Spain.

Based on our research evidence, the above cultural competence barriers at clinical, structural and organisational levels should be addressed in parallel with other health system deficiencies, such as saturation, excess of demand, insufficient time per patient [65], in order to improve access to SRH services and reduce health inequities among immigrant populations. Additionally, we observed the need of developing further studies exploring and evaluating the effectiveness of new roles, such as community health agents, who not only provide intercultural mediation services, but also reach, promote and facilitate access to the health system among the most vulnerable immigrants within their communities.

This study has several methodological strengths and limitations. It is noteworthy to mention that the researchers’ social position (e.g. gender, age, race, immigration status) and personal characteristics and experiences affect the research process [66]. In this research, four authors have Moroccan or Pakistani origin, two authors were immigrant either in the Catalan/Spanish context or another foreign country and two authors were not immigrants and of Catalan origin. This diversity in backgrounds and experiences offered both insider- and outsider-perspectives on the topic, enhancing the richness in the research process and its outcomes. The insider-perspective enabled a deeper comprehension of participants’ perceptions and interactions with the Catalan health system, allowing an accurate reflection in their viewpoints. Simultaneously, the outsider-perspectives from the non-immigrant authors in the Catalan/Spanish context provided a professional viewpoint that broadened the perspective beyond personal experiences. The inclusion of immigrant women’s with and without language barriers and healthcare providers’ perspectives is also an important strength of this study, as it facilitated an in depth analysis where linguistic and cultural differences were confronted in a constructive way.

Regarding the study limitations, it is important to mention that the inclusion of only two national groups limits the generalizability of the findings to other immigrant experiences and cultural backgrounds, although it serves as a valuable case study. Additionally, the small sample size of healthcare professionals, who primarily work in areas with high concentration of immigrant populations, may not capture the perspectives from those working in areas where immigrants are less represented.

## Conclusion

This study emphasizes significant cultural competence barriers within the Catalan health system that hinder immigrant patient-provider relationship, potentially increasing disparities in accessing SRH services in Catalonia, Spain. Addressing these barriers requires a fair deployment of intercultural mediators, clear guidelines to help manage cultural challenges, the formalization of cultural competence training for healthcare professionals, and a commitment to increase workforce diversity. These steps are crucial to mitigating the impact of language barriers and cultural differences on communication between immigrant patients and providers, thereby improving access to quality care. Health systems across Europe, including the Catalan health system, are urged to take a proactive role and implement on-the-ground actions aiming to improve cultural competence and ensure an equitable provision of quality care for immigrant populations.

## Electronic supplementary material

Below is the link to the electronic supplementary material.

Supplementary Material 1

Supplementary Material 2

Supplementary Material 3

## Data Availability

The datasets used and/or analysed during the current study are available from the corresponding author on reasonable request.

## Abbreviations

FGD: Focus group discussion
RA: Research assistant
SRH: Sexual and reproductive health
SSI: Semi-structured interview
STI: Sexually transmitted infection
