# Supplementary material for: Exploring cultural competence barriers in the primary care sexual and reproductive health centres in Catalonia, Spain: perspectives from immigrant women and healthcare providers
Source: Int J Equity Health. 2024 Oct 9;23:206. doi: 10.1186/s12939-024-02290-5 (PMC11465850; doi:10.1186/s12939-024-02290-5)
Supplement: Supplementary file 1 — Supplementary Material 1 [file 12939_2024_2290_MOESM1_ESM.pdf]

Excerpt of the codebook used for analysis of the FGDs and SSIs with immigrant women from Morocco and Pakistan

**Key topic: Immigrant women's experiences with the Catalan health system**		
**Themes/Sub-themes**	**Code description**	**Examples of supporting quotes**
Catalan vs Moroccan and Pakistani health systems	Women's general perceptions of the Catalan health system in comparison with the health systems in Morocco and Pakistan.	*MG001- Well, the good thing is that the health system is free of charge, you don't pay… In Morocco, there are doctors, thanks to God, but if you have money, you get cure; if you don't have money, you don't get cure.*<br>*PG102- I think here it's better than the Pakistan's health system. Here [in Spain] it is well organised, systematized and doctors have good control of the patients. I personally liked it, they give you one medicine if you're sick and if it doesn't work then they'll change it.*
Familiarity with the health system<br>- Booking appointment<br>- Use of primary and emergency services	Knowledge of women on how to navigate the Catalan health system, including booking appointments in person and through the digital health platform 'La Meva Salut'; familiarity with the different levels of care and information about SRH services and patients' rights and responsibilities.	*MC08- Yes, to book an appointment with the specialist, you need to visit first your general practitioner in the primary care facility and then, this doctor has to refer you to the gynaecologist or another specialist, because these specialists work in the hospital.*<br>*PC03- To get an appointment with the doctor you can go in person or you can arrange the appointment by phone as well, but it's much easier to go straightaway to the emergency services.*
Difficulties to get appointments	Narratives about women's issues to arrange appointments in the Catalan health system, such as 'gatekeepers', long waiting lists.	*PG202- You don't get appointments easily. Doctors are on vacation and substitute doctors are not available. And you get an appointment after four months. And after four months you forget what your problem was. Seriously, it has happened to me, especially with gynaecologists. I think they don't have many gynaecologists.*
Patient-provider relationship<br>- Trusting and caring professionals<br>- Short and impersonal consultations<br>- Negative attitudes towards immigrants<br>- Exchange of information	Women's feelings, perceptions and descriptions of their interactions with the health system staff, including healthcare providers (doctors, nurses, community health workers), clinic receptionists, pharmacists, social workers, intercultural mediators.	*MG205- La verdad que me tocó un sirio y me gustó, me explicó y me gustó porque… aunque sea nuestro idioma daryja, no es sirio, pero lo he entendido y me ha entendido. Y en todos los hospitales encuentras… depende del médico… mi madre que tiene un problema de columna tiene un médico, para mí, racista. No se comporta. No me gusta ni ir a él. No te dedica tiempo, yo le digo que tiene dolor y él me dice que bueno, ves al médico de cabecera y ellos te darán medicamentos, porque es su médica y le podrá dar medicamento, pero no te dedica tiempo. Rápido te pasa.*<br>*MG202- A few years ago, I couldn't speak Spanish and I couldn't find anyone who could accompany me to the midwife*

		consultation. My husband worked in Portugal. Perhaps it was my responsibility to bring someone and be able to answer her questions… She spoke to me very aggressively. I can't forget it.<br>*PC05- Generally they [healthcare providers] are kind, but there are receptionists in the health centres who don't have any sensitivity when they attend a person who can't speak properly in Spanish or Catalan. Instead of being empathetic and try to communicate or ask for help, they just ask 'what are you saying?', then, this person won't come back. This is what happens with Pakistani women, they prefer to stay at home or wait to go to the gynaecologist in their country and pay, rather than face these unpleasant situations. Honestly, we prefer to pay [back home] and avoid taking an appointment here.*
Communication barriers<br>- Language limitations<br>- Family and friends as translators<br>- Intercultural mediators<br>- Diversity of the workforce	Women's accounts regarding their communication barriers within the Catalan health system. This includes patients' language limitations and their implications, coping strategies and proposed solutions.	*PC01- Many (Pakistani) women don't speak Spanish properly and they depend on their husbands' schedules to go to the doctor, and husbands work the whole day from Monday to Friday, so that's why women don't want to come.*<br>*MC06- I prefer to go to the doctor with someone who I know to help me with translation. I think I'm more comfortable. Before my neighbour used to come with me.*<br>*PC08- Not at all. I try to manage with the bit Spanish I know, in other case I try to use gestures to make the doctor understand what I want to say. I try my best to make them understand what I want to say using my hands, body language.*
Difficulties to get a same sex healthcare provider	Women's perceptions and personal experiences requesting a same sex provider in the primary care SRH centres in Barcelona. This includes when they requested a female doctor (e.g. what was the reaction of the provider? was it difficult? how is the process?)	*MC01- There is a Moroccan woman in my neighbourhood who requested a female doctor in her surgery and up to now she has been having troubles just because she wanted a female doctor (…) Sometimes the receptionists in the health centre don't like that an immigrant woman requests a female doctor…they think she needs to accept what is available, that's it, they think that immigrants do not have rights and can't demand anything.*<br>*PG203- I had an experience here, a woman who recently came from Pakistan, she requested a female doctor, and the receptionist started to argue and say: 'that's the problem with you Pakistanis' and then the woman didn't get an appointment until three months later and the scan was in another hospital far*

		from there and the husband asked to get the appointment in their health centre but the receptionist ignored it.
Mistrust in the health system	Women's perceptions and experiences of getting treated within the Catalan health system (e.g. trust in the healthcare providers' diagnosis and prescriptions).	*PG202 - basically here initially small things make a big disease, and they prolong it, their analysis is weak, actually it is weak, it is very important to emphasize on it, they just give you paracetamol or ibuprofen and after 10 or 15 days when they realize that you're getting worse instead of getting better, then they give you antibiotic, only one.*<br>*PG305- In Pakistan they give us medication and we immediately get cured by that, but here they do everything like blood tests and other tests but it takes a lot of time and even after tests we don't get our reports, we don't get what is the problem, I've many issues, I've a guilty (zed) here and they said if we will cut this then you'll get 10 more so we can't cut this one. "Drink water, eat salad and walk". All day what I do is walk as I've two grandchildren at home, so I've to go for grocery, take them out, etc, I don't sit at home, I don't walk like jogging but I didn't get anything special here like Pakistan [she referred to treatment].*
Traumatic experiences	Women's narratives describing traumatic experiences within the Catalan health system.	*PC03- I was pregnant and they did the test [Pap smear] and I started bleeding like when you have your period. I was scared. I told them that I was going to lose my baby, because I was six months pregnant, I think. They shouldn't have done the test. They told me that it was normal and there was not reason to be worried, but they did the test and they shouldn't have done it.*
