# Supplementary material for: Exploring cultural competence barriers in the primary care sexual and reproductive health centres in Catalonia, Spain: perspectives from immigrant women and healthcare providers
Source: Int J Equity Health. 2024 Oct 9;23:206. doi: 10.1186/s12939-024-02290-5 (PMC11465850; doi:10.1186/s12939-024-02290-5)
Supplement: Supplementary file 2 — Supplementary Material 2 [file 12939_2024_2290_MOESM2_ESM.pdf]

Excerpt of the codebook used for analysis of the KIIs with healthcare providers

**Key topic: Healthcare providers' experiences providing care to immigrant patients in the Catalan health system**		
**Themes/Sub-themes**	**Concepts**	**Example of supporting quotes**
Communication barriers<br>- Language limitations<br>- Cultural differences<br>- Strategies to overcome language barriers<br>- Translator volunteers<br>- Intercultural mediators<br>- Translation resources	Healthcare providers' challenges in their interaction with immigrant patients, for instance, when explaining a diagnosis, treatment or giving a follow up appointment, or when examining them.	*KI02- We manage with our body language; it's true that using gestures, we and Moroccan women understand each other quite well, but non-verbal communication with Pakistani women is more difficult, because their body language, gestures are different to ours. I'm always afraid of doing a gesture that can appear offensive (female midwife).*<br>*KI03- We used to have some pictograms in different languages, but what I usually do is to give the information with drawings and sometimes with images as they help them to visualize a little bit what and where is the health issue (female gynaecologist).*<br>*KI11- The other day a man came to my consult with his wife who had a vaginal problem and he didn't know how to express the problem. He told me that she had pain "in the secret", and I couldn't understand what 'the secret' was. He couldn't explain the problem in a different way. In some cultures, men find it difficult to speak with a woman about these issues (female midwife).*<br>*KI09- In the ASSIR unit [primary care SRH centre] we have two auxiliary nurses, one from Pakistan and one from Morocco. They are usually working in the maternity ward, but sometimes they go to the primary care units to help with translations, but it's not their role, they are auxiliary nurses…" (male gynaecologist).*<br>*KI02- The availability of intercultural mediators is very important for us. Ten years ago we had the possibility of these services, it was a project funded by the Caixa Foundation, they trained mediators from different cultures, and then unfortunately this service disappeared in almost all primary healthcare centres" (female midwife)*

		KI12- The difficulty I found in the beginning is that the Moroccan interpreter translated according to her beliefs, which was a handicap. For example, a young girl with an unwanted pregnancy came and I asked whether she wanted to continue with the pregnancy or interrupt it, and the Moroccan interpreter 'jumped' into the conversation with a smile telling me 'we [Muslims] don't interrupt pregnancies'. I needed to tell her that she only had to translate without judgements or giving opinions. After some time and with more experience, we solved this situation. It's important to train the interpreters [referring to intercultural mediators] and make sure they understand their role (female midwife).<br>*KI09- It's the slowest thing in the world! I have 15 minutes per visit! It takes ages until they find an available translator, you can't wait for this. We need more resources, like mediators. I manage with materials and forms translated into various languages" (male gynaecologist).*
Patient-provider relationship<br>- Frustration and exhaustion<br>- Time pressure<br>- Role of the husband and family<br>- Exchange of information<br>- Informed consent<br>- Lack of privacy and confidentiality	Healthcare providers' feelings, concerns and experiences when providing healthcare services to immigrant patients.	*KI05- These visits are difficult and require more time… The time factor is like a sword stuck in your back. Sometimes you need to spend more time with these women to make sure they understand why we are doing these tests [cervical cancer screening tests]. (…) After so many years, I'm at a point where I feel exhausted (..) I'm constantly gesticulating to try to reach and communicate with these women; it's exhausting (female midwife).*<br>*KI02- Many times I have the impression that it's the husband who makes the decisions for them. For example, I ask him to translate and he just makes a very short translation and then he basically orders her to take off her underwear for the vaginal examination. But I need her to understand me, I need him to*

		translate well and her to make the decision" (female midwife).<br>*KI05- ...It's really hard, but it's like this. If you have some empathy with the husband, this woman will come back, because we can't build rapport with some women, they don't speak any word in Spanish (female midwife).*<br>*KI11- Many times we cannot explain much more because otherwise we had to start giving them a class of anatomy in fifteen minutes (...) But it's true that sometimes I simplify a little bit when it comes to cervical cancer prevention and we just say that we'll check that 'everything is ok' (female midwife).*<br>*KI11- Of course, there are some specific issues we can't address. For example, we have a questionnaire to screen all pregnant women for domestic violence. If her partner comes always to the visit with her, we can't address this. It's true that you can observe the relationship between them, behaviours during the visit, etc, but it's difficult (female midwife)*
Cultural competence skills<br>- Training<br>- Diversity of the workforce	Providers' self-assessment of intercultural communication skills, perceived availability of training in cultural competence for health staff and their views on diversity in the workplace.	*KI06- I miss in our university medical studies this cross-cultural perspective, because the view is very 'Western' and we need to keep in mind that in other cultures people understand health and wellbeing in different ways. I think the experience working with these populations gives you the knowledge, you learn from immigrant patients about their habits, cultural beliefs, etc. Empathy and cultural sensitivity are the basics (female general practitioner)*<br>*KI02- We had a Chinese gynaecologist and at that time Chinese patients started to come to the clinics like never before (female midwife).*
Perceived discrimination due to sex of the provider	Providers' opinions about the possibility to choose the sex of the	*KI09- You have the right to have a healthcare provider, to be attended, but you can't choose whether the doctor is white or black, or whether the doctor is female or male, because*

	healthcare provider in the public health system	*it's considered discriminatory. In the private health system, yes, you can choose whatever you want, but in the public health system, not (male gynaecologist).*<br>*KI10- They can't officially request a female doctor just like you can't request a white gynae or a heterosexual gynae … It'd bother me if someone doesn't want me to visit her just because I'm a woman. I know that there is a cultural barrier, but it annoys me that a professional can be judged because of his/her gender (…) Obviously, if the woman has suffered a sexual aggression, all of us would be empathetic [to her preference for a female provider] (female gynaecologist).*
Universal health care (UHC)	Knowledge about immigrant patients' rights to access healthcare services in the Catalan health system	KI06- *"not all health staff know the law and the bureaucratic circuits of the health system. There is confusion among the administrative staff and there are situations in which they may deny health services to immigrants because of lack of knowledge" KI06 (female general practitioner).*
