# Supplementary material for: Exploring cultural competence barriers in the primary care sexual and reproductive health centres in Catalonia, Spain: perspectives from immigrant women and healthcare providers
Source: Int J Equity Health. 2024 Oct 9;23:206. doi: 10.1186/s12939-024-02290-5 (PMC11465850; doi:10.1186/s12939-024-02290-5)
Supplement: Supplementary file 3 — Supplementary Material 3 [file 12939_2024_2290_MOESM3_ESM.pdf]

Triangulation of the study findings using the cultural competence framework proposed by Betancourt et al., 2003.

	Synthesized themes and sub-themes	Quotes from KII with providers	Quotes from FGD & SSI with immigrant women
**Clinical barriers**			
	**1. Lack of language skills**	*"Sometimes I see the husband explaining her wife what the doctor is saying, but not all, some just say a few things because they think the wife doesn't need to know everything" KI04 (Pakistani community health worker)*<br><br>*"The first and most important thing is to make sure that women understand the information we provide, because we often give them the information through a third party [referring to the husband] who is not transmitting the information properly. It's crucial to have a mediator or a person committed who can explain all of this, otherwise women won't come to get screened" KI05 (female midwife)*<br><br>*"They usually come with their own resources, like a mobile phone and someone on the other side of the phone line who does the translation, or a friend, or a neighbour… and sometimes they bring me a paper with the health issues or questions written by their partner (in Spanish) and I respond to them by hand on the same paper. I also explain to them that they need to come with a person who can translate, otherwise it's very complicated for her to understand the treatment" KI12 (female midwife)*<br><br>*"We manage with our body language; it's true that using gestures, we and Moroccan women understand each other quite well, but non-verbal communication with Pakistani women is more difficult, because their body language, gestures are different to ours. I'm always afraid of doing a gesture that can appear offensive" KI02 (female midwife)*<br><br>*"We used to have some pictograms in different languages, but what I usually do is to give the information with drawings and sometimes with images as they help them to*	*"Many (Pakistani) women don't speak Spanish properly and they depend on their husbands' schedules to go to the doctor, and husbands work the whole day from Monday to Friday, so that's why women don't want to come" PC01 (Pakistani woman)*<br><br>*"I prefer to go to the doctor with someone who I know to help me with translation. I think I'm more comfortable. Before my neighbour used to come with me" MC06 (Moroccan woman)*

		visualize a little bit what and where is the health issue" KI03 (female gynaecologist)<br><br>*"I have learned a couple of words in Arabic and I usually use them with Maghrebi women. They can see that I make efforts to facilitate communication and I create some empathy" KI05 (female midwife).*<br><br>*"I speak a little French, sometimes I ask Moroccan patients if they speak French, if they say 'yes', then we manage in French" KI09 (male gynaecologist).*	
	**2. Perceived discriminatory attitudes**	*"You have the right to have a healthcare provider, to be attended, but you can't choose whether the doctor is white or black, or whether the doctor is female or male, because it's considered discriminatory. In the private health system, yes, you can choose whatever you want, but in the public health system, not" KI09 (male gynaecologist)*<br><br>*"They can't officially request a female doctor just like you can't request a white gynae or a heterosexual gynae … It'd bother me if someone doesn't want me to visit her just because I'm a woman. I know that there is a cultural barrier, but it annoys me that a professional can be judged because of his/her gender (…) Obviously, if the woman has suffered a sexual aggression, all of us would be empathetic [to her preference for a female provider]" KI10 (female gynaecologist)*	*"A few years ago, I couldn't speak Spanish and I couldn't find anyone who could accompany me to the midwife consultation. My husband worked in Portugal. Perhaps it was my responsibility to bring someone and be able to answer her questions… She spoke to me very aggressively. I can't forget it" MG202 (Moroccan woman)*<br><br>*"Generally they [healthcare providers] are kind, but there are receptionists in the health centres who don't have any sensitivity when they attend a person who can't speak properly in Spanish or Catalan. Instead of being empathetic and try to communicate or ask for help, they just ask 'what are you saying?', then, this person won't come back. This is what happens with Pakistani women, they prefer to stay at home or wait to go to the gynaecologist in their country and pay, rather than face these unpleasant situations. Honestly, we prefer to pay [back home] and avoid taking an appointment here" PC05 (Pakistani woman).*<br><br>*"There is a Moroccan woman in my neighbourhood who requested a female doctor in her surgery and up to now she has been having troubles just because she wanted a female doctor (…) Sometimes the receptionists in the health centre don't like that an immigrant woman requests a female doctor…they think she needs to accept what is available,*

			that's it, they think that immigrants do not have rights and can't demand anything" MC01 (Moroccan woman)<br><br>*"I had an experience here, a woman who recently came from Pakistan, she requested a female doctor, and the receptionist started to argue and say: 'that's the problem with you Pakistanis' and then the woman didn't get an appointment until three months later and the scan was in another hospital far from there and the husband asked to get the appointment in their health centre but the receptionist ignored it" PG203 (Pakistani woman)*
	**3. Time pressure in the provision of care**	*"These visits are difficult and require more time… The time factor is like a sword stuck in your back. Sometimes you need to spend more time with these women to make sure they understand why we are doing these tests [cervical cancer screening tests]. (…) After so many years, I'm at a point where I feel exhausted (..) I'm constantly gesticulating to try to reach and communicate with these women; it's exhausting" KI05 (female midwife)*<br><br>*"Sometimes they don't understand the treatment, some of them don't know how to navigate the health system or how to get the public health insurance (…) Some have low education and many times they come with medical consultations, basic issues, such as a headache, that other people wouldn't be worried about [meaning additional burden]" KI11 (female midwife)*	*"Despite my broken Spanish, I try to explain the doctor my problems in detail, but she always cuts me off and doesn't give me enough time to express myself. It's like they work under lot of pressure, and since they have lots of patients, they attend you quickly" MC11 (Moroccan woman)*
	**4. Cultural taboos**	*"A woman came with her 9 years old son to help her to translate. I asked the child to ask his mum the date of her last menstruation and the child told me that he couldn't ask this kind of question to his mum… They are difficult situations, because culturally women don't talk about these topics in front of specific family members [referring to males and children]. For me, it was an important barrier because I need this information" KI02 (female midwife)*	*"If you are pregnant, our religion doesn't allow us to get abortion, you cannot ask the doctor for abortion services… you are pregnant and you need to accept it" MC05 (Moroccan woman)*

		"For example, I had a patient who rejected to be examined because it was Ramadan and she told me it was not allowed during Ramadan. She came three weeks later for the examination" KI03 (female gynaecologist)<br><br>*"The other day a man came to my consult with his wife who had a vaginal problem and he didn't know how to express the problem. He told me that she had pain "in the secret", and I couldn't understand what 'the secret' was. He couldn't explain the problem in a different way. In some cultures, men find it difficult to speak with a woman about these issues" KI11 (female midwife)*<br><br>*"Many times we cannot explain much more because otherwise we had to start giving them a class of anatomy in fifteen minutes (…) But it's true that sometimes I simplify a little bit when it comes to cervical cancer prevention and we just say that we'll check that 'everything is ok'" KI11 (female midwife)*	
	**5. Limited patient informed consent and confidentiality**	*Many times I have the impression that it's the husband who makes the decisions for them. For example, I ask him to translate and he just makes a very short translation and then he basically orders her to take off her underwear for the vaginal examination. But I need her to understand me, I need him to translate well and her to make the decision" KI02 (female midwife).*<br><br>*"…It's really hard, but it's like this. If you have some empathy with the husband, this woman will come back, because we can't build rapport with some women, they don't speak any word in Spanish" KI05 (female midwife)*<br><br>*"Of course, there are some specific issues we can't address. For example, we have a questionnaire to screen all pregnant women for domestic violence. If her partner comes always to the visit with her, we can't address this. It's true that you*	

		can observe the relationship between them, behaviours during the visit, etc, but it's difficult" KI11 (female midwife)<br><br>*"When a mediator is available we can ask the partner to get out or the woman can come alone another day and then it's easier to address these issues [e.g. STIs, vaginal problems] with the presence of a mediator"* KI11 (female midwife)	
**Structural barriers**			
	**6. Limited availability of intercultural mediators**	*"The availability of intercultural mediators is very important for us. Ten years ago we had the possibility of these services, it was a project funded by the Caixa Foundation, they trained mediators from different cultures, and then unfortunately this service disappeared in almost all primary healthcare centres"* KI02 (female midwife)<br><br>*"In the ASSIR unit [primary care SRH centre] we have two auxiliary nurses, one from Pakistan and one from Morocco. They are usually working in the maternity ward, but sometimes they go to the primary care units to help with translations, but it's not their role, they are auxiliary nurses…"* KI09 (male gynaecologist).<br><br>*"The difficulty I found in the beginning is that the Moroccan interpreter translated according to her beliefs, which was a handicap. For example, a young girl with an unwanted pregnancy came and I asked whether she wanted to continue with the pregnancy or interrupt it, and the Moroccan interpreter 'jumped' into the conversation with a smile telling me 'we [Muslims] don't interrupt pregnancies'. I needed to tell her that she only had to translate without judgements or giving opinions. After some time and with more experience, we solved this situation. It's important to train the interpreters [referring to intercultural mediators] and make sure they understand their role"* KI12 (female midwife)	

		"The women didn't want him [referring to a male intercultural mediator] to be in the medical consultation, because he was a man. They felt uncomfortable with a male interpreter and they were worried that he could know someone in their community" KI02 (female midwife).	
	**7. Limited and underused translation services and materials**	*"They put you in contact with a health translator and then the interpreter gives you simultaneous translation via phone. I think it's helpful and ensures anonymity"* KI02 (female midwife)<br><br>*"It's the slowest thing in the world! I have 15 minutes per visit! It takes ages until they find an available translator, you can't wait for this. We need more resources, like mediators. I manage with materials and forms translated into various languages"* KI09 (male gynaecologist)	
	**8. Lack of adequate cultural competence training for health staff**	*"I miss in our university medical studies this cross-cultural perspective, because the view is very 'Western' and we need to keep in mind that in other cultures people understand health and wellbeing in different ways. I think the experience working with these populations gives you the knowledge, you learn from immigrant patients about their habits, cultural beliefs, etc. Empathy and cultural sensitivity are the basics"* KI06 (female general practitioner)<br><br>*"not all health staff know the law and the bureaucratic circuits of the health system. There is confusion among the administrative staff and there are situations in which they may deny health services to immigrants because of lack of knowledge"* KI06 (female general practitioner).	
**Organisational barriers**			
	**9. Low minority representation in the healthcare workforce**	*"We had a Chinese gynaecologist and at that time Chinese patients started to come to the clinics like never before"* KI02 (female midwife).	*"He was a Syrian doctor and I liked it, he explained me in Arabic and even though my local language is Darija, I understood him"* MG205 (Moroccan woman).
	**10. Low minority representation in leadership roles**	*Field observations*	*Field Observations*
